# Association between False Lumen Origin of Segmental Arteries and Postoperative Spinal Cord Injury in Acute Type A Aortic Dissection

**DOI:** 10.5761/atcs.oa.26-00121

**Published:** 2026-08-27

**Authors:** Kotaro Mukasa, Shinichiro Abe, Tomoya Furukawa, Musashi Tsuda, Soichi Asano

**Affiliations:** Department of Cardiovascular Surgery, Chiba Cardiovascular Center, Ichihara, Chiba, Japan

**Keywords:** acute type A aortic dissection, spinal cord injury, segmental artery, false lumen, contrast-enhanced computed tomography

## Abstract

**Purpose:**

We examined whether false lumen-origin segmental arteries (SAs) were associated with postoperative spinal cord injury (SCI) after surgery for acute Stanford type A aortic dissection (ATAAD).

**Methods:**

We reviewed 201 consecutive patients who underwent open repair for ATAAD between January 2008 and February 2026. After prespecified exclusions, 124 patients were analyzed, including 83 with evaluable contrast-enhanced computed tomography for detailed SA assessment.

**Results:**

Postoperative SCI occurred in 9 patients (7.3%), who comprised the SCI group; the remaining 115 comprised the non-SCI group. Total arch replacement and frozen elephant trunk use were numerically more frequent in the SCI group. In the detailed cohort, the number of false lumen-origin SAs was higher in the SCI group at T3–T7 (7.2 ± 3.0 vs. 3.9 ± 3.1, P = 0.042) and T8–L2 (10.3 ± 2.7 vs. 5.5 ± 4.2, P = 0.005). The corresponding proportions were also higher at T3–T7 (77.5% vs. 45.2%, P = 0.047) and T8–L2 (75.0% vs. 41.9%, P = 0.010).

**Conclusion:**

In this exploratory single-center cohort, false lumen-origin SAs were associated with postoperative SCI in unadjusted analyses. Further studies are needed to determine their value for preoperative risk stratification.

## Introduction

Acute Stanford type A aortic dissection (ATAAD) is a life-threatening disease in which the risk of death increases rapidly after onset, and it requires prompt diagnosis and surgical treatment. Although treatment outcomes have improved in recent years, postoperative neurologic complications remain an important issue.^1)^ In particular, spinal cord injury (SCI), although less frequent than cerebral infarction, is a serious complication that substantially affects survival, activities of daily living, and long-term quality of life.^2)^

SCI after surgery for ATAAD has mainly been discussed in relation to surgical invasiveness and operative strategy. In particular, total arch replacement (TAR) using a frozen elephant trunk (FET) is considered a potential contributor to SCI.^3–5)^ Previous reports on SCI after FET have identified a more distal stent graft landing level as a risk factor for SCI.^5)^ However, SCI can also occur in patients who do not undergo FET or extensive arch replacement, suggesting that some cases cannot be fully explained by intercostal artery coverage by the FET alone.

Spinal cord perfusion does not depend solely on a single artery of Adamkiewicz. Rather, it is maintained by an extensive collateral network that includes the intercostal arteries, lumbar arteries, subclavian arteries, internal iliac arteries, and paraspinal muscular vascular networks.^6)^ According to this concept, blood flow input from multiple segmental arteries (SAs) is important for maintaining spinal cord perfusion. On the other hand, the artery of Adamkiewicz is a major inflow vessel to the anterior spinal artery system, and it has been reported to arise most frequently around T8–L1 or T8–L2.^7)^ Therefore, T8–L2 can be considered an anatomically important region for spinal cord perfusion. In addition, SAs at the upper thoracic levels may also contribute to spinal cord blood flow through the collateral network, and the origin status of SAs at T3–T7 cannot be ignored.

In ATAAD, when the dissection extends from the descending thoracic aorta to the abdominal aorta, SAs may arise from either the true lumen or the false lumen. Even if false lumen-origin SAs are perfused by false lumen flow before surgery, their perfusion may decrease postoperatively because of reduced false lumen flow after resection of the proximal entry tear. Therefore, in patients with many SAs arising from the false lumen on preoperative computed tomography (CT), the reserve capacity of spinal cord perfusion may be reduced after surgery, which may increase susceptibility to SCI.

Recently, several reports have suggested that false lumen origin of SAs, particularly around the thoracolumbar junction, is associated with SCI.^4,8,9)^ However, many of these studies have focused on FET, and limited data are available on the detailed evaluation of SA origin using preoperative contrast-enhanced CT and its association with SCI across the overall spectrum of surgery for ATAAD. In this study, we investigated the association between SA origin on preoperative contrast-enhanced CT and the occurrence of postoperative SCI in patients who underwent open aortic graft replacement for ATAAD. We evaluated whether the number and proportion of false lumen-origin SAs were associated with postoperative SCI.

## Materials and Methods

### Study design and patients

This was a single-center retrospective observational study. We reviewed 201 consecutive patients who underwent open aortic graft replacement for ATAAD at our institution between January 2008 and February 2026. Patients with preoperative paraplegia, intraoperative dissection caused by arterial perfusion, dissection not extending to the descending aorta, intramural hematoma without ulcer-like projection, or inadequate preoperative contrast-enhanced CT assessment were excluded. Intramural hematoma with ulcer-like projection was not excluded in this study because a certain degree of flow space or communication within the false lumen may have been present. Finally, 124 patients were included in the analysis. Patients who developed postoperative SCI were classified into the SCI group, and those who did not were classified into the non-SCI group. In addition, a detailed analysis of segmental artery origin was performed in 83 patients in whom the origin of the SAs could be assessed in detail on preoperative contrast-enhanced CT. Assessment of SAs on CT was performed independently by 2 cardiovascular surgeons. Discrepant assessments were adjudicated by a third cardiovascular surgeon. Branches that could not be classified were categorized as undetermined after discussion. This study was approved by the institutional review board of our hospital, approval number 153. Consent for the use of clinical data for research was obtained in accordance with institutional policy.

### Definitions

SCI was defined as newly developed postoperative paraplegia or paraparesis that was not present preoperatively. The diagnosis was always made in consultation with a neurologist. No patient in the present cohort had paraplegia before surgery. Patients who underwent preoperative electrocardiogram-gated contrast-enhanced CT with a slice thickness of 1 mm or less were considered to have detailed CT assessment. In patients with detailed preoperative CT assessment, the origin of each segmental artery at each vertebral level was classified as arising from the true lumen or the false lumen. Each segmental artery was assessed separately on the right and left sides and classified as true lumen-origin or false lumen-origin. SAs for which the origin could not be determined on imaging were classified as undetermined.

### Surgical technique

Moderate hypothermic circulatory arrest with a target bladder temperature of 26°C and selective antegrade cerebral perfusion was routinely used for cerebral protection. When severe aortic atheroma was present or thrombus was suspected within the supra-aortic branch vessels, retrograde cerebral perfusion was initiated first and was then converted to antegrade cerebral perfusion after aortotomy. Femoral artery cannulation was the first choice for arterial inflow, with additional axillary artery cannulation used according to the dissection pattern. Surgery was performed through a standard median sternotomy, and cardiopulmonary bypass was established with right atrial drainage. Myocardial protection was achieved with retrograde cold blood cardioplegia through the coronary sinus. After circulatory arrest was established, the ascending aorta was opened, and the location of the primary entry tear was identified. The extent of graft replacement was determined according to a tear-oriented strategy, with the basic aim of resecting the primary entry tear. Resection of the primary entry tear was achieved in all patients in the study cohort. When TAR was performed, a FET was generally used. However, FET was not used in the early period of the cohort because it was not commercially available at that time.

Cerebrospinal fluid drainage was performed in all patients once SCI was diagnosed. Prophylactic cerebrospinal fluid drainage was not performed in patients without SCI. In a retrospective review, no patient received steroid therapy for SCI. After the onset of SCI, systolic blood pressure was maintained at 120 mmHg or higher, and hemoglobin levels were managed with a target of at least 12 g/dL.

### Endpoints

The primary endpoint was the occurrence of postoperative SCI. Secondary endpoints were comparisons of patient characteristics, preoperative dissection morphology, operative procedures, and perioperative outcomes between the SCI and non-SCI groups. In patients in whom detailed SA assessment was feasible, the associations between postoperative SCI and the number and proportion of false lumen-origin SAs at T3–T7 and T8–L2 were evaluated. The vertebral levels were categorized in this manner for the reasons described in the Introduction.

### Statistical analysis

Continuous variables are presented as mean ± standard deviation or median [interquartile range]. Categorical variables are presented as numbers and percentages. For comparisons between the 2 groups, Welch’s t test or the Mann–Whitney U test was used for continuous variables, and Fisher’s exact test or the chi-square test was used for categorical variables. A P value <0.05 was considered statistically significant. Because the number of postoperative SCI events was small, multivariable analysis was not performed to avoid overfitting. Therefore, the logistic regression analyses were interpreted as exploratory and unadjusted. Univariable logistic regression analyses were additionally performed to estimate odds ratios and 95% confidence intervals for clinically relevant variables associated with postoperative SCI. Continuous operative time variables were analyzed per 10-minute increase. Age was analyzed per 10-year increase, body mass index per 1 kg/m^2^ increase, lowest body temperature per 1°C increase, and false lumen-origin SA proportion per 10% increase.

The primary analysis was based on univariable comparisons and descriptive analyses. Analyses were performed using R software, version 4.5.0 (R Core Team).

## Results

### Patient selection and incidence of SCI

**Fig. 1:**
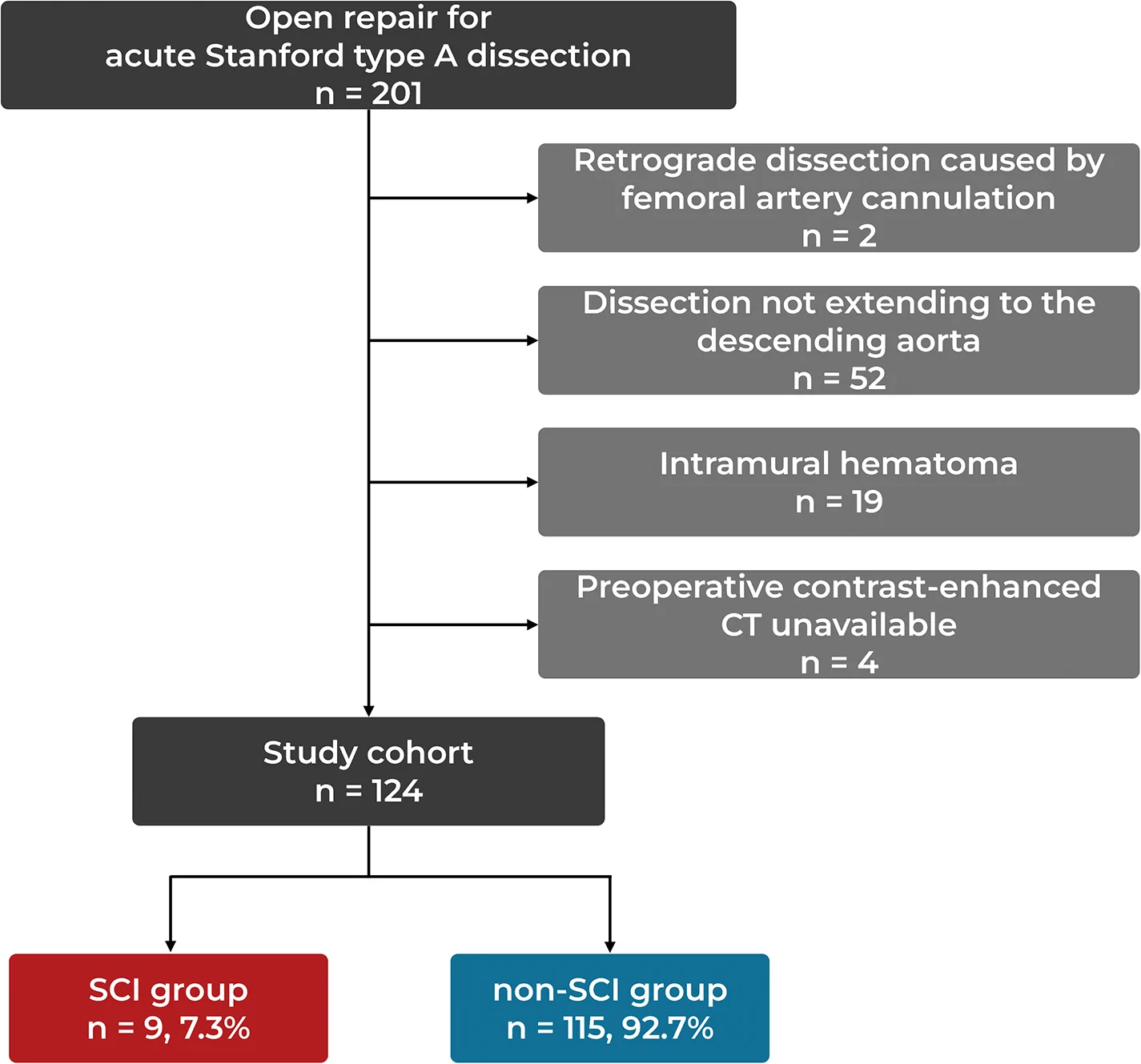
The study cohort. Flowchart showing the exclusion process and final classification of patients into the SCI and non-SCI groups. SCI, spinal cord injury

Among 201 patients who underwent open aortic graft replacement for ATAAD during the study period, 2 patients with new dissection caused by intraoperative arterial perfusion, 52 patients in whom the dissection did not extend to the descending aorta, 19 patients with intramural hematoma without ulcer-like projection, and 4 patients in whom preoperative contrast-enhanced CT could not be adequately assessed were excluded. The final study cohort included 124 patients. Postoperative SCI occurred in 9 patients, with an incidence of 7.3%. The remaining 115 patients were classified into the non-SCI group (**Fig. 1**). Detailed assessment of segmental artery origin was possible in 83 patients, including 6 patients in the SCI group and 77 patients in the non-SCI group.

### Patient characteristics

Patient characteristics are shown in **Table 1**. The mean age of the overall cohort was 64.8 ± 11.4 years, and 68 patients (54.8%) were male. There were no significant differences between the SCI and non-SCI groups in age, sex, body mass index, chronic obstructive pulmonary disease, diabetes mellitus, dialysis, or estimated glomerular filtration rate. Age was 62.4 ± 12.1 vs. 65.0 ± 11.4 years (P = 0.559), the proportion of male patients was 55.6% vs. 54.8% (P = 1.000), and body mass index was 23.6 ± 5.4 vs. 22.8 ± 3.7 kg/m^2^ (P = 0.655) in the SCI and non-SCI groups, respectively. Unless otherwise indicated, values are presented in this order throughout the Results section.

**Table 1:** Patient characteristics

Variables	All	SCI	Non-SCI	P value
	124 (100)	9 (7.3)	115 (92.7)	
Age, y	64.8 ± 11.4	62.4 ± 12.1	65.0 ± 11.4	0.559
Sex, male	68 (54.8)	5 (55.6)	63 (54.8)	1.000
Body mass index, kg/m^2^	22.8 ± 3.8	23.6 ± 5.4	22.8 ± 3.7	0.655
COPD	1 (0.8)	0 (0.0)	1 (0.9)	1.000
Diabetes mellitus	3 (2.4)	1 (11.1)	2 (1.7)	0.204
Dialysis	1 (0.8)	0 (0.0)	1 (0.9)	1.000
eGFR, mL/min/1.73 m^2^	55.6 ± 17.8	58.1 ± 17.0	55.4 ± 17.9	0.678
Cardiac tamponade	13 (10.5)	0 (0.0)	13 (11.3)	0.595
Shock	21 (16.9)	0 (0.0)	21 (18.3)	0.355
Resuscitation before surgery	3 (2.4)	0 (0.0)	3 (2.6)	1.000
Heritable aortic disease	5 (4.0)	0 (0.0)	5 (4.3)	1.000
Redo	2 (1.6)	0 (0.0)	2 (1.8)	1.000
Previous aortic surgery	1 (0.8)	0 (0.0)	1 (0.9)	1.000
Primary entry				0.223
Aortic root	1 (0.8)	0 (0.0)	1 (0.9)	
Ascending aorta	61 (49.2)	2 (22.2)	59 (51.3)	
Aortic arch	50 (40.3)	7 (77.8)	43 (37.4)	
Descending aorta	4 (3.2)	0 (0.0)	4 (3.5)	
Unknown	8 (6.5)	0 (0.0)	8 (7.0)	
False lumen status				1.000
Patent false lumen	120 (96.8)	9 (100.0)	111 (96.5)	
IMH with ULP	4 (3.2)	0 (0.0)	4 (3.5)	
Organ malperfusion	41 (33.1)	1 (11.1)	40 (34.8)	0.269
Coronary	2 (1.6)	0 (0.0)	2 (1.7)	1.000
Cerebral	22 (17.7)	0 (0.0)	22 (19.1)	0.359
Superior mesenteric artery	5 (4.0)	0 (0.0)	5 (4.3)	1.000
Renal artery	8 (6.5)	0 (0.0)	8 (7.0)	1.000
Lower extremity	15 (12.1)	1 (11.1)	14 (12.2)	1.000

Values are mean ± standard deviation or number (%).

COPD, chronic obstructive pulmonary disease; eGFR, estimated glomerular filtration rate; IMH with ULP, intramural hematoma with ulcer-like projection; SCI, spinal cord injury

There were also no significant differences in preoperative status, including cardiac tamponade, shock, preoperative resuscitation, heritable aortic disease, redo surgery, or previous aortic surgery. The primary entry tended to be more frequently located in the aortic arch in the SCI group, although the difference was not statistically significant. Aortic arch entry was observed in 77.8% vs. 37.4%, with an overall P value of 0.223. Regarding false lumen status, most patients had a patent false lumen, with rates of 100.0% vs. 96.5% (P = 1.000).

Preoperative organ malperfusion was observed in 33.1% of the overall cohort and in 11.1% vs. 34.8% of patients (P = 0.269). There were no significant differences in coronary, cerebral, superior mesenteric artery, renal artery, or lower extremity malperfusion.

### Operative factors

Operative variables are shown in **Table 2**. Regarding the extent of graft replacement, TAR was numerically more frequent in the SCI group than in the non-SCI group (66.7% vs. 35.7%), although the difference did not reach statistical significance. Among the 9 patients in the SCI group, 2 underwent ascending aortic replacement, 1 underwent partial arch replacement, and 6 underwent TAR. Of the 6 patients who underwent TAR, 3 underwent TAR using FET. FET use was also numerically more frequent in the SCI group than in the non-SCI group (33.3% vs. 20.0%, P = 0.395).

**Table 2:** Operative details

Variables	All	SCI	Non-SCI	P value
	124 (100)	9 (7.3)	115 (92.7)	
Extension of graft replacement				0.270
Ascending aorta	50 (40.3)	2 (22.2)	48 (41.7)	
Partial arch	27 (21.8)	1 (11.1)	26 (22.6)	
Total arch	47 (37.9)	6 (66.7)	41 (35.7)	
TAR with FET	26 (21.0)	3 (33.3)	23 (20.0)	0.395
Cannulation site				
Axillary	56 (45.2)	3 (33.3)	53 (46.1)	0.511
Femoral	114 (91.9)	9 (100.0)	105 (91.3)	1.000
Ascending aorta	1 (0.8)	0 (0.0)	1 (0.9)	1.000
Apex	1 (0.8)	0 (0.0)	1 (0.9)	1.000
Cerebral perfusion strategy				1.000
SCP	122 (98.4)	9 (100.0)	113 (98.3)	
Combined	2 (1.6)	0 (0.0)	2 (1.7)	
Operation time, min	481.6 ± 146.5	470.2 ± 97.8	482.4 ± 149.9	0.737
CPB time, min	286.5 ± 89.7	281.4 ± 59.7	286.9 ± 91.9	0.804
Aortic cross-clamp time, min	155.3 ± 49.9	158.4 ± 41.1	155.0 ± 50.6	0.819
Circulatory arrest time, min	71.1 ± 20.8	81.3 ± 33.3	70.3 ± 19.5	0.353
Cerebral perfusion time, min	140.4 ± 82.2	169.8 ± 79.4	138.1 ± 82.3	0.279
Lowest body temperature, °C	23.6 ± 2.0	24.0 ± 1.6	23.5 ± 2.0	0.430
Concomitant procedure				
AVR	6 (4.8)	0 (0.0)	6 (5.2)	1.000
Bentall	6 (4.8)	0 (0.0)	6 (5.2)	1.000
CABG	4 (3.2)	0 (0.0)	4 (3.5)	1.000
RBC transfusion, units	11.4 ± 10.4	11.8 ± 5.1	11.4 ± 10.7	0.835

Values are mean ± standard deviation or number (%).

TAR, total arch replacement; FET, frozen elephant trunk; CPB, cardiopulmonary bypass; SCP, selective cerebral perfusion; AVR, aortic valve replacement; CABG, coronary artery bypass grafting; SCI, spinal cord injury; RBC, red blood cells

In univariable logistic regression analysis, the odds ratio for postoperative SCI was 3.61 for TAR and 2.00 for FET use, although the confidence intervals were wide. Arch entry was associated with postoperative SCI, with an odds ratio of 5.86. Circulatory arrest time and cerebral perfusion time showed odds ratios of 1.22 and 1.05 per 10-minute increase, respectively. These results are summarized in **Table 3**.

**Table 3:** Univariable logistic regression analysis for postoperative SCI

Variable	Cohort	n	SCI events	Unit	OR	95% CI	P value
Age	Overall cohort	124	9	Per 10 years	0.83	0.47–1.47	0.523
Male sex	Overall cohort	124	9	Yes vs. no	1.03	0.26–4.04	0.964
Body mass index	Overall cohort	121	9	Per 1 kg/m^2^	1.06	0.89–1.26	0.522
Arch entry	Overall cohort	124	9	Yes vs. no	5.86	1.16–29.50	0.032
Organ malperfusion	Overall cohort	124	9	Yes vs. no	0.23	0.03–1.94	0.179
Total arch replacement	Overall cohort	124	9	Yes vs. no	3.61	0.86–15.20	0.080
FET use	Overall cohort	124	9	Yes vs. no	2.00	0.46–8.61	0.352
Operation time	Overall cohort	124	9	Per 10 min	0.99	0.95–1.04	0.809
Cardiopulmonary bypass time	Overall cohort	124	9	Per 10 min	0.99	0.92–1.07	0.859
Aortic cross-clamp time	Overall cohort	123	9	Per 10 min	1.01	0.89–1.15	0.843
Circulatory arrest time	Overall cohort	124	9	Per 10 min	1.22	0.94–1.59	0.135
Cerebral perfusion time	Overall cohort	124	9	Per 10 min	1.05	0.97–1.14	0.270
Lowest body temperature	Overall cohort	123	9	Per 1°C	1.14	0.79–1.64	0.498
T3–T7							
False lumen-origin SA	Detailed CT cohort	83	6	Per 1 artery	1.41	1.04–1.91	0.028
False lumen-origin SA proportion	Detailed CT cohort	82	6	Per 10%	1.41	1.02–1.96	0.039
T8–L2							
False lumen-origin SA	Detailed CT cohort	83	6	Per 1 artery	1.35	1.05–1.73	0.018
False lumen-origin SA proportion	Detailed CT cohort	83	6	Per 10%	1.49	1.05–2.10	0.025

CI, confidence interval; CT, computed tomography; FET, frozen elephant trunk; OR, odds ratio; SA, segmental artery; SCI, spinal cord injury

FET-specific operative details are shown in **Supplementary Table 1**. Among 26 patients who underwent FET implantation, the mean FET diameter was 27.6 ± 3.3 mm and the mean FET length was 85.8 ± 20.4 mm. The distal landing level was T7 in all 3 FET patients who developed SCI. Among patients with an identifiable distal landing level, 1 patient had a distal landing level distal to T8, and this patient did not develop SCI. Device types are summarized in **Supplementary Table 1**. Information on distal perfusion during FET implantation was not systematically available.

### Postoperative outcomes

Postoperative outcomes are shown in **Table 4**. In-hospital mortality occurred in 13 patients (10.5%) and was observed in 11.1% vs. 10.4% (P = 1.000). Symptomatic stroke occurred in 44.4% vs. 32.2% (P = 0.476), and re-exploration for bleeding was performed in 22.2% vs. 6.1% (P = 0.129). Renal replacement therapy was required in 33.3% vs. 20.0% (P = 0.395), and hemodialysis was initiated in 0.0% vs. 4.3% (P = 1.000).

**Table 4:** Postoperative outcomes

Variables	All	SCI	Non-SCI	P value
	124 (100)	9 (7.3)	115 (92.7)	
In-hospital mortality	13 (10.5)	1 (11.1)	12 (10.4)	1.000
Symptomatic stroke	41 (33.1)	4 (44.4)	37 (32.2)	0.476
Re-exploration for bleeding	9 (7.3)	2 (22.2)	7 (6.1)	0.129
Renal replacement therapy	26 (21.0)	3 (33.3)	23 (20.0)	0.395
New hemodialysis	5 (4.0)	0 (0.0)	5 (4.3)	1.000
Tracheostomy	9 (7.3)	3 (33.3)	6 (5.2)	0.018
DANE	13 (10.5)	0 (0.0)	13 (11.3)	0.595
ICU stay, days	5.1 [3.1, 10.0]	28.8 [8.0, 29.3]	4.9 [3.1, 8.6]	0.001
Hospital stay, days	30.0 [23.0, 47.2]	53.0 [48.0, 73.0]	28.0 [22.0, 42.0]	0.004

Values are presented as mean ± standard deviation, number (%), or median [interquartile range].

DANE, distal anastomotic new entry; ICU, intensive care unit; SCI, spinal cord injury

Tracheostomy was performed in 33.3% vs. 5.2% (P = 0.018) and was significantly more frequent in the SCI group. The intensive care unit stay was 28.8 days [8.0, 29.3] vs. 4.9 days [3.1, 8.6] (P = 0.001), and the postoperative hospital stay was 53.0 days [48.0, 73.0] vs. 28.0 days [22.0, 42.0] (P = 0.004). Both were significantly longer in the SCI group. These variables were evaluated as postoperative outcomes and may reflect the clinical consequences of SCI or greater overall postoperative morbidity.

### Detailed assessment of segmental artery origin

**Fig. 2:**
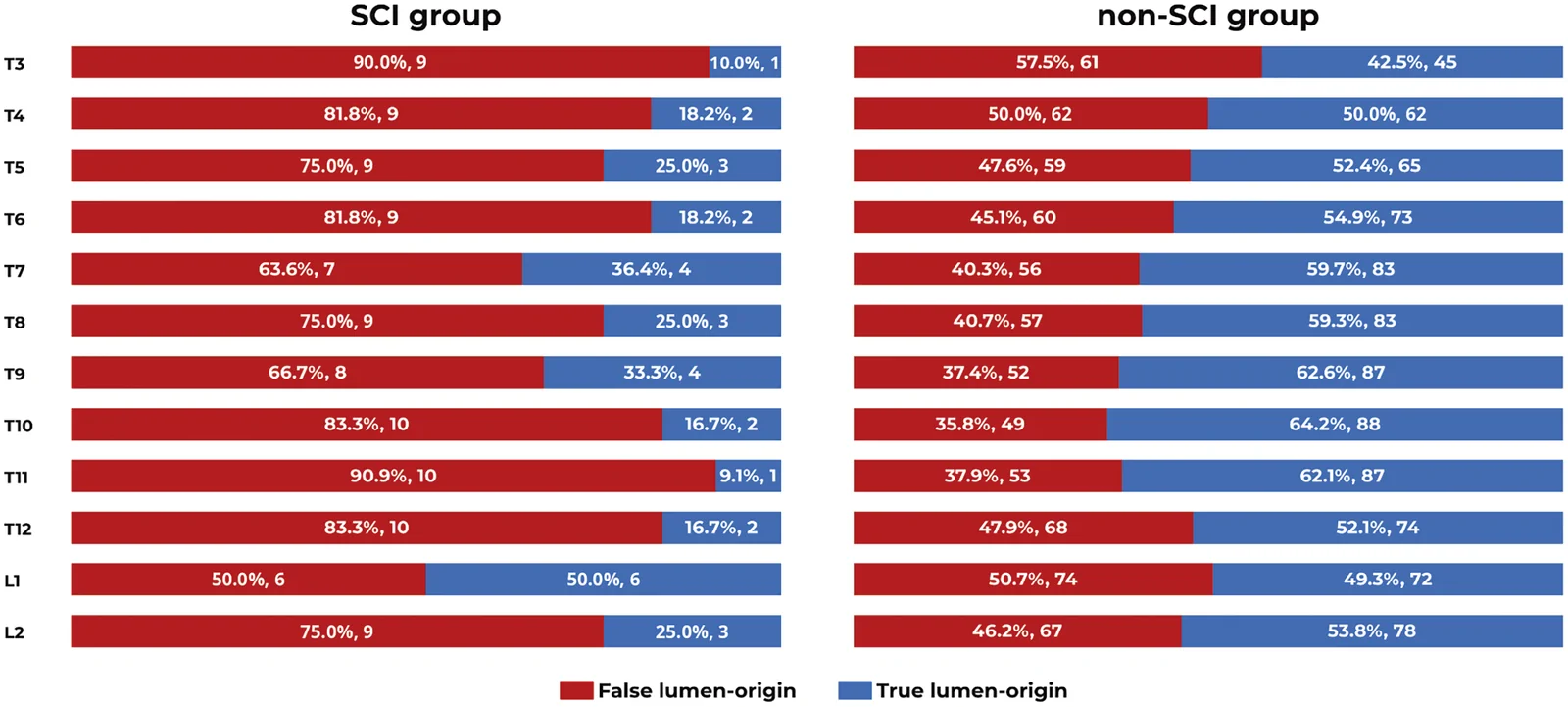
Distribution of segmental artery origin according to vertebral level. Stacked bar charts showing the proportions and numbers of false lumen-origin and true lumen-origin SAs at each vertebral level in the SCI and non-SCI groups. Values within the bars indicate the percentage and number of evaluable SAs. Undetermined SAs were excluded from the denominator. SAs, segmental arteries; SCI, spinal cord injury

Segmental artery origin at T3–T7 and T8–L2 was analyzed in 83 patients in whom detailed assessment using preoperative contrast-enhanced CT was feasible. The results are summarized in **Table 5**, and the distribution of SA origins by vertebral level is shown in **Fig. 2**.

**Table 5:** Distribution of segmental artery origin in patients with detailed preoperative contrast-enhanced CT assessment

Variables	All	SCI	Non-SCI	P value
	83 (100)	6 (7.2)	77 (92.8)	
T3–T7				
Evaluable SA, n	8.2 ± 2.4	9.2 ± 1.3	8.1 ± 2.5	0.129
False lumen-origin SA, n	4.1 ± 3.2	7.2 ± 3.0	3.9 ± 3.1	0.042
True lumen-origin SA, n	4.1 ± 2.9	2.0 ± 2.5	4.3 ± 2.8	0.082
False lumen-origin SA proportion, %	47.5 ± 33.7	77.5 ± 30.3	45.2 ± 33.0	0.047
T8–L2				
Evaluable SA, n	12.9 ± 2.1	13.8 ± 0.4	12.8 ± 2.1	0.002
False lumen-origin SA, n	5.8 ± 4.3	10.3 ± 2.7	5.5 ± 4.2	0.005
True lumen-origin SA, n	7.1 ± 4.1	3.5 ± 2.9	7.4 ± 4.1	0.021
False lumen-origin SA proportion, %	44.3 ± 31.2	75.0 ± 21.1	41.9 ± 30.7	0.010

Values are presented as mean ± standard deviation.

SA, segmental artery; CT, computed tomography; SCI, spinal cord injury

At T3–T7, the number of evaluable SAs was 9.2 ± 1.3 vs. 8.1 ± 2.5 (P = 0.129). The number of false lumen-origin SAs was 7.2 ± 3.0 vs. 3.9 ± 3.1 (P = 0.042), which was significantly higher in the SCI group. The number of true lumen-origin SAs was 2.0 ± 2.5 vs. 4.3 ± 2.8 (P = 0.082), showing a tendency to be lower in the SCI group. The proportion of false lumen-origin SAs was 77.5 ± 30.3% vs. 45.2 ± 33.0% (P = 0.047), which was significantly higher in the SCI group.

At T8–L2, the number of evaluable SAs was 13.8 ± 0.4 vs. 12.8 ± 2.1 (P = 0.002). The number of false lumen-origin SAs was 10.3 ± 2.7 vs. 5.5 ± 4.2 (P = 0.005), which was significantly higher in the SCI group. The number of true lumen-origin SAs was 3.5 ± 2.9 vs. 7.4 ± 4.1 (P = 0.021), which was significantly lower in the SCI group. The proportion of false lumen-origin SAs was 75.0 ± 21.1% vs. 41.9 ± 30.7% (P = 0.010), which was significantly higher in the SCI group.

In univariable logistic regression analysis in the detailed CT cohort, the odds ratios for postoperative SCI were 1.41 per additional false lumen-origin SA at T3–T7 and 1.35 per additional false lumen-origin SA at T8–L2. The odds ratios were 1.41 per 10% increase in the false lumen-origin SA proportion at T3–T7 and 1.49 per 10% increase at T8–L2. These results are shown in **Table 3**.

### Comparison between patients with and without detailed CT assessment

Patient characteristics, dissection morphology, operative variables, and postoperative outcomes were compared between patients with and without detailed preoperative CT assessment, as shown in **Supplementary Table 2**. Age, sex, body mass index, estimated glomerular filtration rate, and the frequency of organ malperfusion were similar between the 2 cohorts.

Cardiac tamponade was less frequent in the detailed CT cohort than in the non-detailed CT cohort (4.8% vs. 22.0%, P = 0.009), as was preoperative shock (12.0% vs. 26.8%, P = 0.046). The distribution of the primary entry location differed between the cohorts (P = 0.013).

The extent of graft replacement differed between the cohorts (P <0.001). Partial arch replacement or TAR was more frequent, whereas ascending aortic replacement was less frequent, in the detailed CT cohort. FET use was more frequent in the detailed CT cohort (27.7% vs. 7.3%, P = 0.009). Cerebral perfusion time was longer (158.8 ± 78.8 vs. 103.3 ± 76.9 min, P <0.001), and the lowest body temperature was lower (23.2 ± 1.9°C vs. 24.4 ± 1.9°C, P <0.001), in the detailed CT cohort. However, the incidence of postoperative SCI (7.2% vs. 7.3%, P = 1.000) and in-hospital mortality (9.6% vs. 12.2%, P = 0.757) did not differ between the cohorts.

## Discussion

The main finding of this study was that patients who developed postoperative SCI had a higher number and proportion of SAs originating from the false lumen on preoperative contrast-enhanced CT. Two observations merit discussion. First, this association was observed in a cohort not limited to FET procedures, and univariable analyses suggested a possible association between false lumen-origin SAs and postoperative SCI. These findings support the possibility that false lumen-origin SAs may reflect preexisting anatomical vulnerability of spinal cord perfusion, rather than merely serving as a surrogate marker for direct intercostal artery coverage by FET. Second, this association was observed not only at T8 to L2, which is anatomically important as the common origin region of the artery of Adamkiewicz, but also at T3 to T7. This broader distribution supports the concept that postoperative SCI after ATAAD repair may be influenced by vulnerability of the entire spinal cord collateral network, rather than by isolated compromise of the thoracolumbar segment alone. However, because the number of SCI events was small, all logistic regression analyses were unadjusted, and TAR and FET use were numerically more frequent in the SCI group, operative strategy and dissection morphology cannot be excluded as potential confounders. Therefore, the present study does not establish that false lumen-origin SAs are independently associated with SCI.

Postoperative SCI after ATAAD surgery has mainly been discussed in relation to FET.^3–5)^ FET can promote remodeling of the descending aorta and may reduce late aorta-related events.^10)^ Therefore, it has recently been widely used as part of an aggressive arch repair strategy for ATAAD. Conversely, stent graft coverage of the descending aorta by FET can directly interrupt blood flow to the intercostal arteries and has therefore been recognized as a potential risk factor for SCI. In the meta-analysis by Preventza et al., SCI occurred with a certain frequency after FET, and longer stent grafts or coverage beyond T8 were reported to be associated with increased SCI risk.^5)^ Perioperative blood pressure fluctuation and the presence of atheroma have also been considered important risk factors.^3,11)^

However, the present findings highlight the limitations of attributing SCI solely to intercostal artery coverage by FET. In this cohort, only 3 of the 9 patients with SCI underwent TAR with FET, while the remaining patients developed SCI after procedures without FET. Although TAR with FET is likely an important risk factor for SCI, as previously reported, the occurrence of SCI after procedures without FET in the present cohort highlights the need to investigate additional contributing factors.

This interpretation is consistent with recent reports regarding the association between SA origin and SCI. Kodama et al. reported that SAs originating from the false lumen were associated with SCI.^9)^ Dong et al. showed that false lumen-dependent SAs were associated with SCI in patients with acute DeBakey type I dissection undergoing FET.^8)^ Wei et al. demonstrated that a posterior false lumen at T9 to L2 was associated with paraplegia after FET.^4)^ In addition, a large-scale study by Wei et al. identified SA involvement, prolonged hypothermic circulatory arrest time, and low intraoperative mean arterial pressure as risk factors for SCI after ATAAD surgery.^2)^ The present study complements these findings by showing that, even in a broader population not limited to FET cases, the preoperative SA origin pattern assessed by contrast-enhanced CT may be associated with postoperative SCI after open prosthetic graft replacement with entry tear resection for ATAAD.

A possible mechanism is postoperative alteration of false lumen perfusion. In ATAAD extending to the descending aorta or further downstream, SAs may arise from either the true lumen or the false lumen. Before surgery, even SAs originating from the false lumen may maintain a certain degree of perfusion through false lumen flow via the proximal entry tear or distal re-entry tears. However, once the proximal entry tear is resected, false lumen pressure and flow may change, and partial or complete thrombosis of the false lumen may progress.^12)^ Although this change is desirable from the perspective of aortic remodeling, it may reduce perfusion of SAs that had depended on false lumen flow. Thus, in patients with many SAs originating from the false lumen preoperatively, the operation itself may become a trigger for reduced inflow into the spinal cord collateral network. However, because sufficient postoperative contrast-enhanced CT data were not available, this mechanism could not be validated radiographically and therefore remains hypothetical.

The observed association between false lumen origin of SAs and SCI at T8–L2 is anatomically plausible. The artery of Adamkiewicz often arises from the lower thoracic to upper lumbar levels, and SAs in this region may serve as major inflow vessels to the anterior spinal artery system.^7)^ In the present study, both the number and proportion of SAs originating from the false lumen at T8–L2 were significantly higher in the SCI group, whereas the number of SAs originating from the true lumen was significantly lower. This finding suggests that patients with SCI may have had less stable true lumen inflow to the spinal cord at T8–L2 and were therefore vulnerable to postoperative reduction in false lumen flow.

It is also important that the number and proportion of SAs originating from the false lumen at T3–T7 were higher in the SCI group. Spinal cord perfusion does not depend solely on a single artery of Adamkiewicz. It is maintained by an extensive collateral network consisting of the intercostal arteries, lumbar arteries, subclavian arteries, internal iliac arteries, and paraspinal muscular vascular networks.^6)^ The observed difference at T3–T7 may indicate that upper thoracic SAs also contribute to spinal cord perfusion as part of the collateral network, or that the widespread presence of SAs originating from the false lumen reflects overall vulnerability of spinal cord perfusion. However, these findings should be interpreted cautiously because multiple SA variables were compared across 2 anatomic regions, increasing the possibility of false-positive findings. This concern is particularly relevant to the borderline associations observed at T3–T7. In addition, a dissection morphology in which SAs at T8–L2 extensively originate from the false lumen may also tend to involve more proximal SAs in the same manner.

Clinically, SA origin on preoperative contrast-enhanced CT may represent a candidate imaging marker for postoperative SCI after ATAAD surgery. In ATAAD, rapid surgical decision-making is required to prioritize survival, and detailed identification of the artery of Adamkiewicz is not realistic in all patients. However, when thin-slice contrast-enhanced CT is available, evaluating whether SAs originate from the true or false lumen may help identify patients at high risk for SCI before surgery. In particular, in patients with a posterior false lumen and many SAs originating from the false lumen, more stringent management should be considered. This may include avoidance of intraoperative hypotension, maintenance of sufficient hemoglobin concentration and cardiac output, shortening of lower body circulatory arrest time, early restoration of distal aortic perfusion, careful selection of FET length and distal landing level, and rigorous early postoperative neurological assessment.^3,13)^ However, the present study was limited by its small sample size, and further studies with larger cohorts are warranted.

The comparison between patients with and without detailed CT assessment provides important context for interpreting the present findings. The incidence of postoperative SCI and in-hospital mortality was similar between the 2 cohorts. However, the detailed CT cohort differed from the non-detailed CT cohort in hemodynamic presentation, dissection morphology, and several operative variables, including the extent of graft replacement, FET use, cerebral perfusion time, and temperature management. These differences indicate that the detailed CT cohort represented a selected subgroup and may not have been fully representative of the overall study population.

### Limitations

This study has several limitations. First, this was a single-center retrospective study with a limited number of patients who developed postoperative SCI. Therefore, multivariable adjustment was not performed to avoid overfitting. Second, detailed SA assessment was limited to patients with adequate preoperative contrast-enhanced CT. Because the detailed CT cohort differed from the non-detailed CT cohort in several characteristics, selection bias cannot be excluded. Third, interobserver variability in SA classification was not assessed. Fourth, the study spanned 18 years, during which CT protocols, operative strategies, device availability, spinal cord protection, and postoperative management may have changed. The first FET procedure in this cohort was performed in 2018. Because of the limited number of SCI events, an era-stratified sensitivity analysis was not performed. Finally, postoperative SCI was analyzed as a binary outcome, and its severity, timing of onset, recovery course, and detailed neurologic phenotype were not included in the present analysis.

## Conclusion

In this exploratory single-center cohort, a higher number and proportion of false lumen-origin SAs were associated with postoperative SCI in unadjusted analyses. Larger multicenter studies are needed to validate this association and determine whether SA-origin assessment can contribute to preoperative risk stratification.

## Supplementary Materials

Supplementary Table 1 Operative details in patients who underwent frozen elephant trunk implantation

Supplementary Table 2 Comparison between patients with and without detailed preoperative contrast-enhanced computed tomography assessment

## Declarations

### Data availability

The data that support the findings of this study are available from the corresponding author upon reasonable request.
