# Supplementary material for: Association between False Lumen Origin of Segmental Arteries and Postoperative Spinal Cord Injury in Acute Type A Aortic Dissection
Source: Ann Thorac Cardiovasc Surg. 2026 Aug 27;32(1):26-00121. doi: 10.5761/atcs.oa.26-00121 (PMC13521652; doi:10.5761/atcs.oa.26-00121)
Supplement: Supplementary file 1 [file atcs-32-1-26-00121-s001.pdf]

Supplementary Table 1. Operative details in patients who underwent frozen elephant trunk implantation

Variable	All FET cases	SCI	non-SCI
	26 (100)	3 (11.5)	23 (88.5)
FET diameter, mm	27.6 ± 3.3	32.3 ± 4.6	27.0 ± 2.6
21 mm	1 (3.8)	0 (0.0)	1 (4.3)
23 mm	1 (3.8)	0 (0.0)	1 (4.3)
24 mm	1 (3.8)	0 (0.0)	1 (4.3)
25 mm	5 (19.2)	0 (0.0)	5 (21.7)
26 mm	1 (3.8)	0 (0.0)	1 (4.3)
27 mm	6 (23.1)	1 (33.3)	5 (21.7)
29 mm	6 (23.1)	0 (0.0)	6 (26.1)
31 mm	3 (11.5)	0 (0.0)	3 (13.0)
35 mm	2 (7.7)	2 (66.7)	0 (0.0)
FET length, mm	85.8 ± 20.4	80.0 ± 17.3	86.5 ± 21.0
60 mm	8 (30.8)	1 (33.3)	7 (30.4)
90 mm	13 (50.0)	2 (66.7)	11 (47.8)
100 mm	1 (3.8)	0 (0.0)	1 (4.3)
120 mm	4 (15.4)	0 (0.0)	4 (17.4)
Distal landing level			
T5	3 (11.5)	0 (0.0)	3 (13.0)
T6	6 (23.1)	0 (0.0)	6 (26.1)
T7	11 (42.3)	3 (100.0)	8 (34.8)
T8	2 (7.7)	0 (0.0)	2 (8.7)
T9	1 (3.8)	0 (0.0)	1 (4.3)
Unknown	3 (11.5)	0 (0.0)	3 (13.0)
Device type			
FROZENIX	19 (73.1)	2 (66.7)	17 (73.9)
FROZENIX 4-branched	3 (11.5)	1 (33.3)	2 (8.7)
FROZENIX partial ET	3 (11.5)	0 (0.0)	3 (13.0)
Thoraflex	1 (3.8)	0 (0.0)	1 (4.3)

FET, frozen elephant trunk; SCI, spinal cord injury.

FROZENIX (Japan Lifeline Co., Ltd., Tokyo, Japan)

Thoraflex Hybrid (Terumo Aortic, Inchinnan, UK)

Supplementary Table 2. Comparison between patients with and without detailed preoperative contrast-enhanced computed tomography assessment

Variable	All	Detailed CT	Non-detailed CT	P value
	124 (100)	83 (66.9)	41 (33.1)	
Baseline characteristics				
Age, y	64.8 ± 11.4	65.4 ± 11.0	63.6 ± 12.2	0.432
Body mass index, kg/m²	22.8 ± 3.8	22.8 ± 3.8	22.8 ± 3.8	0.931
eGFR, mL/min/1.73 m²	55.6 ± 17.8	56.8 ± 17.6	52.7 ± 18.2	0.270
Male sex	68 (54.8)	47 (56.6)	21 (51.2)	0.702
Cardiac tamponade	13 (10.5)	4 (4.8)	9 (22.0)	0.009
Shock	21 (16.9)	10 (12.0)	11 (26.8)	0.046
Resuscitation before surgery	3 (2.4)	1 (1.2)	2 (4.9)	0.254
Organ malperfusion	41 (33.1)	28 (33.7)	13 (31.7)	1.000
Dissection morphology				
Primary entry				0.013
Aortic root or ascending aorta	62 (50.0)	34 (41.0)	28 (68.3)	
Aortic arch or distal arch	50 (40.3)	40 (48.2)	10 (24.4)	
Descending aorta	4 (3.2)	2 (2.4)	2 (4.9)	
Unknown	8 (6.5)	7 (8.4)	1 (2.4)	
Patent false lumen	120 (96.8)	80 (96.4)	40 (97.6)	1.000
Operative variables				
Extent of graft replacement				<0.001
Ascending	50 (40.3)	21 (25.3)	29 (70.7)	
Partial arch	27 (21.8)	23 (27.7)	4 (9.8)	
TAR	47 (37.9)	39 (47.0)	8 (19.5)	
TAR with FET	26 (21.0)	23 (27.7)	3 (7.3)	0.009
Operation time, min	481.6 ± 146.5	480.3 ± 152.2	484.0 ± 135.9	0.892
CPB time, min	286.5 ± 89.7	291.1 ± 90.4	277.3 ± 88.7	0.421
Aortic cross-clamp time, min	155.3 ± 49.9	159.8 ± 54.5	145.9 ± 37.3	0.100
Circulatory arrest time, min	71.1 ± 20.8	70.5 ± 20.8	72.3 ± 20.9	0.653
Cerebral perfusion time, min	140.4 ± 82.2	158.8 ± 78.8	103.3 ± 76.9	<0.001

Lowest body temperature, °C	23.6 ± 2.0	23.2 ± 1.9	24.4 ± 1.9	<0.001
Postoperative outcomes				
Postoperative SCI	9 (7.3)	6 (7.2)	3 (7.3)	1.000
In-hospital mortality	13 (10.5)	8 (9.6)	5 (12.2)	0.757

CT, computed tomography; eGFR, estimated glomerular filtration rate; TAR, Total arch replacement; FET, frozen elephant trunk; CPB, Cardiopulmonary bypass; SCI, spinal cord injury.
